# Digital skin imaging applications, part II: a comprehensive survey of post‐acquisition image utilization features and technology standards

**DOI:** 10.1111/srt.13195

**Published:** 2022-10-01

**Authors:** Mary D. Sun, Jonathan Kentley, Britney W. Wilson, H. Peter Soyer, Clara N. Curiel‐Lewandrowski, Veronica M. Rotemberg, Allan C. Halpern, the ISIC Technique Working Group

**Affiliations:** ^1^ Icahn School of Medicine at Mount Sinai New York New York USA; ^2^ Dermatology Service, Memorial Sloan Kettering New York New York USA; ^3^ Chelsea and Westminster Hospital London UK; ^4^ Rutgers New Jersey Medical School Newark New Jersey USA; ^5^ Dermatology Research Centre Diamantina Institute The University of Queensland Brisbane QLD Australia; ^6^ Division of Dermatology University of Arizona Skin College of Medicine Tucson Arizona USA

**Keywords:** artificial intelligence, clinical imaging, digital tools, mobile applications, quality assurance, teledermatology

## Abstract

**Background:**

Despite the increasing ubiquity and accessibility of teledermatology applications, few studies have comprehensively surveyed their features and technical standards. Importantly, features implemented after the point of capture are often intended to augment image utilization, while technical standards affect interoperability with existing healthcare systems. We aim to comprehensively survey image utilization features and technical characteristics found within publicly discoverable digital skin imaging applications.

**Materials and Methods:**

Applications were identified and categorized as described in Part I. Included applications were then further assessed by three independent reviewers for post‐imaging content, tools, and functionality. Publicly available information was used to determine the presence or absence of relevant technology standards and/or data characteristics.

**Results:**

A total of 20 post‐image acquisition features were identified across three general categories: (1) metadata attachment, (2) functional tools (i.e., those that utilized images or in‐app content to perform a user‐directed function), and (3) image processing. Over 80% of all applications implemented metadata features, with nearly half having metadata features only. Individual feature occurred and feature richness varied significantly by primary audience (*p* < 0.0001) and function (*p* < 0.0001). On average, each application included under three features. Less than half of all applications requested consent for user‐uploaded photos and fewer than 10% provided clear data use and privacy policies.

**Conclusion:**

Post‐imaging functionality in skin imaging applications varies significantly by primary audience and intended function, though nearly all applications implemented metadata labeling. Technical standards are often not implemented or reported consistently. Gaps in the provision of clear consent, data privacy, and data use policies should be urgently addressed.

**Abbreviations**
AI: Artificial intelligence
DTC: Direct‐to‐consumer
EHR: Electronic health record
EMR: Electronic medical record
NHB: Non‐hospital‐based
S&F: Store‐and‐forward
TD: Teledermatology

## 1 INTRODUCTION

Digital dermatology tools are increasingly accessible to patients and providers alike, though sociodemographic challenges remain.^1^ The percentage of U.S. adults who own a smartphone more than doubled from 35% in 2011 to 85% in 2021, with nearly three‐quarters also reporting having desktop or laptop computers and access to in‐home broadband service.^2^ Dermatology‐related smartphone applications (“apps”) grew by over 80% from 2014–2017 and COVID 19‐related policy changes have catalyzed the widespread practice of teledermatology (TD) services.^3^, ^4^, ^5^, ^6^, ^7^ TD is now cited as one of the most common uses of telemedicine and is an active area of clinical investigation.^8^, ^9^, ^10^, ^11^

Skin imaging and image utilization are central to the functionality of many dermatology applications.^12^, ^13^, ^14^ For example, the comparison of lesion images between different timepoints is a useful addition to skin self‐examination tools, while artificial intelligence (AI)‐based diagnostics may augment clinical triage or TD apps. Yet despite the importance and increasing prevalence of these applications, their features and characteristics are not well‐studied.^15^, ^16^ Recent concerns regarding application transparency, data handling, diagnostic accuracy, and medical malpractice have emerged, primarily due to a lack of quality standards and regulatory oversight.^14^, ^17^, ^18^, ^19^, ^20^ The ethical implications of direct‐to‐consumer (DTC) TD have also been called into question.^21^

In this study, we explore image utilization features in skin imaging applications and characterize functional categories, health technology standards, and data handling policies across platforms. Our analysis extends beyond mobile apps to also include web‐based applications, desktop software, and digitals tools that require portable devices.

## 2 METHODS

### 2.1 Identification of mobile/smartphone skin imaging apps

Three reviewers (MS, JK, and BW) conducted keyword searches of the Apple App Store and Google Play Store from January 2021 through February 2021. Searches were performed on three Apple iPhones and a Samsung Galaxy S10 for iOS and Android mobile apps, respectively, with all devices localized to the United States. No language, content, or other search filters were applied. Apps were considered for initial screening if they were associated with at least one of the following search terms: *dermatology*, *TD*, *skin cancer*, *mole*, *melanoma*, *acne*, *eczema*, *psoriasis*, *rosacea*, *rash*, or *hair loss*. Keywords are representative of common dermatologic queries and were adapted from those used in previous studies of dermatology smartphone apps.^3^, ^22^, ^23^, ^24^ Apps that were not available in English, not intended for relevant consumer use in response to dermatologic complaints, or required non‐portable devices were excluded. This search protocol was designed in accordance with the *Quality and Risk of Bias Checklist for Studies that Review Smartphone Applications*.^25^

Reviewers screened app results based on structured fields in the listing, the app description, and accompanying images. Apps were considered if they met the following inclusion criteria: (1) listing categorization indicates medical relevance, (2) in‐app content explicitly references dermatologic disease, and (3) skin imaging and/or skin image upload features are present. The first criterion was met for apps categorized as Medical in the Apple App Store and/or the Google Play Stores, or if the app description was suggestive of clinical management. Common indicators included but were not limited to phrases such as “talk to a doctor,” “provide treatment,” “send a prescription”, and “diagnose.” The second criterion was met if the listing description or in‐app content described identifying, treating, or managing specific dermatologic diseases. The third criterion was met if images in the app listing showed skin imaging capabilities or image upload features, or if the app description or in‐app content referenced uploading images, taking in‐app photos, or submitting a photo/image to a healthcare provider for cutaneous complaints. If the presence of skin imaging features was unclear from the app listing, reviewers downloaded and manually explored the app to determine inclusion.

### 2.2 Identification of web and desktop skin imaging applications

In parallel, reviewers conducted Google Search engine queries for web‐ and desktop‐based skin imaging applications and software tools. To minimize the influence of individual search history, all searches were performed using incognito mode in Google Chrome web browser. Language settings were set to English (United States) and Windows PC devices were localized to the United States. No advanced search filters were used. Given the frequent irrelevance of results returned by web‐based search, reviewers iterated through combinations of various keywords to identify a limited set of applicable search terms: *online dermatology*, *online dermatologist*, *acne prescription*, *eczema prescription*, *hair loss prescription*, and *custom skin prescription* (note: results for *TD* were primarily news articles or academic publications). Due to volume, only the first five pages of search results, or top 70 by Google web rank, were considered. Sponsored results were primarily consumer‐facing TD services and therefore included. Similarly, reviewers then manually explored websites associated with each search result as well as associated media, including web applications and desktop software, and applied the previously described exclusion and inclusion criteria.

### 2.3 Identification of additional mobile apps and digital applications

A small number of additional skin imaging applications described in previous studies of TD^14^, ^26^, ^27^, ^28^, ^29^, ^30^ or recommended by board‐certified dermatologist members of the International Skin Imaging Collaboration (ISIC) were also considered. Recommenders had specialized knowledge of commercial skin imaging services and products. Eleven additional applications were evaluated using the same exclusion and inclusion criteria; of these, six were included.

### 2.4 Assessment of application characteristics, technology standards, and image utilization features

The included set of mobile, web‐based, and desktop applications were loaded onto corresponding smart devices and manually explored by reviewers. Applications were categorized based on their primary audience (consumer‐facing, non‐hospital‐based (NHB) practices, or enterprise/health system) and primary function (educational, clinical triage, store‐and‐forward (S&F) TD, live‐interactive TD, or electronic medical record (EMR) adjunct/clinical imaging storage), as determined by their intended use. Applications that fell under multiple categories were assigned to a primary category based on in‐app content and key functionality. Reviewers also recorded the user type(s) applicable to each app (patient, provider, or both) as well as the availability of an in‐app connection to a licensed healthcare provider. “In‐app connection” was defined as the ability to message, video chat, or send image files to a clinician directly through the application interface.

Descriptive characteristics including application name, developer name, available modalities (iOS, Android mobile, web application, desktop software, portable device), availability (public, private/beta, enterprise‐only), and cost were collected for each application. Reviewers then utilized in‐app content, associated websites and media, and other publicly available sources of information to record information related to technology standards including Health Insurance Portability and Accountability Act (HIPAA) compliance, privacy and data storage policies, filetype compatibility, and integration with EMR/electronic health record (EHR) systems. These information sources, in conjunction with academic literature searches, were also used to determine which applications had been tested in self‐reported or peer‐reviewed studies.

Lastly, reviewers assessed all publicly discoverable in‐app content, tools, and functionality relevant to the use of skin images in each application. Assessments were intended to describe features utilized after image upload and/or the point of image capture. Screenshots that illustrated each feature were recorded, and an inventory of features related to the utilization of skin images was developed (Table 1). Each reviewer independently completed a feature assessment for each application; any features behind a paywall or otherwise not freely available were recorded if they were described in publicly available app descriptions and/or product websites, or if they were demonstrated in app listing screenshots, promotional videos, and/or product demonstrations. Any discrepancies between reviewer assessments were resolved through iterative feature review and consensus discussion.

**TABLE 1:** Definitions of twenty image utilization technique features occurring in included skin imaging applications

**Category**	**#**	**Feature**	**Definition**	**Example**
**Metadata**	1	Free text/structured fields	Option to provide additional information about an image prior to submission, either through free text fields or writing in/selecting options in structured fields	*Free text*: After uploading an image of their skin, the user can write in additional comments in a free text box prior to submission. *Structured fields*: User is asked to answer pre‐written, multiple‐choice questions about the duration, morphology, and evolution of their skin lesion prior to image submission.
	2	Timestamp/temporal labeling	Automatic labeling and/or sorting of images by the date and time uploaded or submitted	When navigating to previously uploaded images, the user sees them arranged in date‐time order with grey timestamps beneath each thumbnail.
	3	2D body map tracking	Ability to identify anatomic location of skin complaint in two‐dimensional space, usually on an avatar	After taking an image of their skin, the user sees a 2D anatomical model and is asked to tap the location closest to that of the real‐life skin lesion.
	4	Image/album labeling	Ability to retitle images or groups of images organized into folders/albums	User selects 3 photos out of the 15 that they have uploaded and moves them to a new in‐app folder, which they title “Rash R Arm March 2020.”
	5	Lesion measurement	Automated (in‐camera or device‐based measurement scale/frame) or guided (with reference object or user‐based estimate) measurement of lesion size	User is instructed to include and identify a reference object (quarter) in their skin image. After the image is uploaded, “2 × 3mm” appears automatically in the size field.
	6	3D body map tracking	Ability to identify anatomic location of skin complaint in three‐dimensional space, usually on an avatar	After taking an image of their skin, the user sees a 3D anatomical model and is asked to rotate, zoom, and then tap on the location closest to that of the real‐life skin lesion.
**Functional tools**	7	AI diagnostic analysis	Application returns some type of diagnostic output with respect to an uploaded image; outputs can be binary, risk class‐based, a list of ranked or unranked diagnoses, and/or a score on a continuous risk scale	After submitting an image to the “AI Analyzer,” the user sees results suggesting that their lesion has a 57.1% chance of malignancy and that they should contact a dermatologist.
	8	Digital share	Ability to share labeled images/image albums, lesion analyses, symptom visualizations, or other in‐app media via email or through other smartphone apps (i.e., text messaging)	When viewing a graph tracking the severity of itch associated with uploaded images over time, the user sees a “Share” button that automatically attaches the PDF to a new email.
	9	Photo reminders	Ability to set and/or customize in‐app reminders to take skin images	A psoriasis patient receives weekly reminders every Saturday to take and upload images of their affected skin.
	10	Compare lesions	Ability to see two or more skin images side by side	After selecting two previously uploaded images and clicking “Compare,” the user sees one on the left side of the screen and the other on the right.
	11	In‐app zoom	Ability to zoom in on or magnify any area of an uploaded image	User selects a photo and pinches in with two fingers to get a magnified view of any area.
	12	File format conversion	In‐app conversion of uploaded skin images between different file types	After uploading a DICOM file to the app interface, the user can choose to download it as a JPG, PNG, or TIFF from within the same application.
**Image processing**	13	Image cropping	Removal of unwanted outer areas from uploaded skin image	After uploading an image, the user trims/removes outer areas representing extraneous background by moving the image borders closer together.
	14	Magnification to resize	Ability to resave a magnified/zoomed in version of a previously uploaded image	After uploading an image, the user pinches in with two fingers to magnify the lesion of concern and saves the magnified version of the image.
	15	Image realignment/orientation	Ability to rotate a previously uploaded image, most commonly to reorient it with a previous image or to facilitate ease of interpretation	After uploading an image, the user hits an arrow button to rotate the image 90°.
	16	Color/contrast correction	Automated or manual ability to adjust color contrast, saturation, filters, and so on, usually with the goal of more accurately representing real‐life appearance	After noticing that their uploaded photo has an orange cast due to harsh lighting, the user adjusts the image contrast and yellow/orange tones using an in‐app color filter.
	17	Image markup	Ability to add text boxes, digital pen markings, or other artificial elements to the image	After uploading an image, the user adds an arrow pointing to the lesion of interest.
	18	Light balance correction	Automated or manual ability to adjust light balance, usually with the goal of more accurately representing real‐life appearance	After taking an image in a shadowy room, the user uses the light adjustment feature to “brighten” the image so that the lesion of interest is more visible.
	19	Obscuration removal	Automated or manual removal of anything covering the lesion/area of interest	After noticing that their bracelet partially obscures their rash in a previously uploaded skin image, the user presses “Remove Obscuration” to replace it with a skin‐colored area.
	20	Background removal	Automated or manual removal of extraneous, non‐skin image background	After noticing a brightly colored shirt in the background of a previously uploaded skin image, the user presses “Remove Background” to replace all non‐skin background with solid grey.

### 2.5 Statistical methods

Logistic regression and Pearson's chi‐square test were performed to assess differences in the occurrence of individual features across primary audience and primary function groups (*p* < 0.05). Where applicable, standardized chi‐square residuals were calculated to assess the relative contribution of specific associations to group differences. Two‐way ANOVA was performed to compare features counts and feature density across application categories (*p* < 0.05). Computational analyses were conducted using R statistical programming software (version 4.02) and figures were generated in R and Venny.^31^, ^32^

## 3 RESULTS

We identified 191 skin imaging applications across mobile, web, and desktop‐based modalities. Of these, 168 (88%) had at least one feature relevant to the use of skin images. A total of 20 features were identified as being relevant to the use of skin images and were classified into three groups based on their general function: (1) metadata, (2) functional tools, and (3) image processing (Table 1). Metadata features added relevant clinical information to skin images, usually in the form of free‐text or structured multiple‐choice fields, to improve lesion monitoring and/or diagnosis. The six metadata features that occurred in our dataset solicited information about anatomic location, duration, time course, clinical symptoms, and lesion size associated with cutaneous complaints. Functional tools features facilitated the ability of users to utilize skin images in clinically relevant ways. The six that occurred in our dataset were intended for a variety of purposes, including time‐lapsed photo comparison, self‐skin exam reminders, and AI‐based diagnostic analysis of uploaded images. Image processing features modified or augmented skin images to improve their usability. Eight features were identified, ranging from image cropping to automated removal of non‐skin image backgrounds.

**FIGURE 1:**
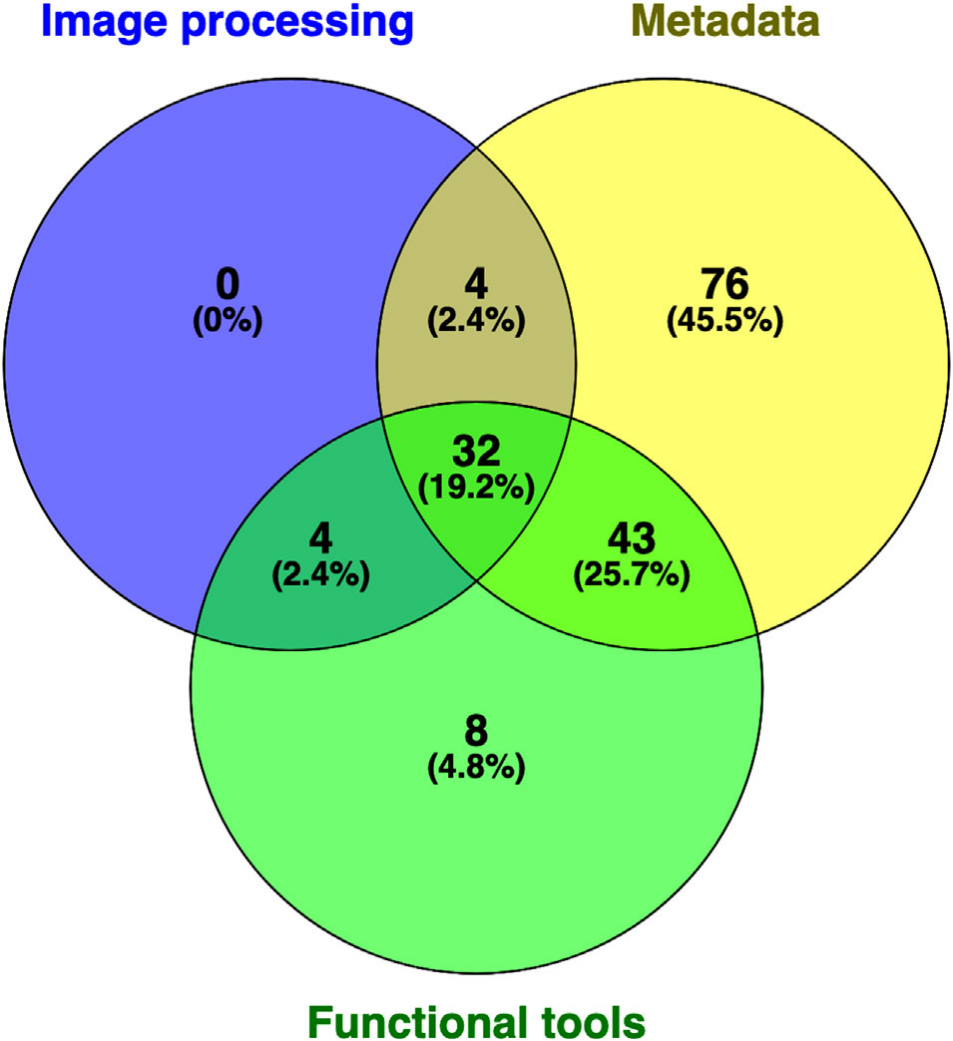
Venn diagram of applications with metadata, functional tools, and image processing features

Metadata features occurred in the largest percentage of applications (81%), followed by functional tools features (46%) and image processing features (21%). Nearly one‐fifth of applications included all three feature types and over a quarter included both metadata and functional tool features. Though almost half of applications contained only metadata features, very few to none contained only functional tools or image processing features, respectively (Figure 1). The three most common features were metadata‐ and functional tools‐related, with 66% of all applications collecting additional text‐based information, 55% generating associated temporal information, and 23% offering some form of AI‐based diagnostic analysis of uploaded images. The image processing‐related features of light balance correction, background removal, and obscuration removal were available only in MatchLab AI, as was file format conversion in Dicompass DICOM Camera.

**FIGURE 2:**
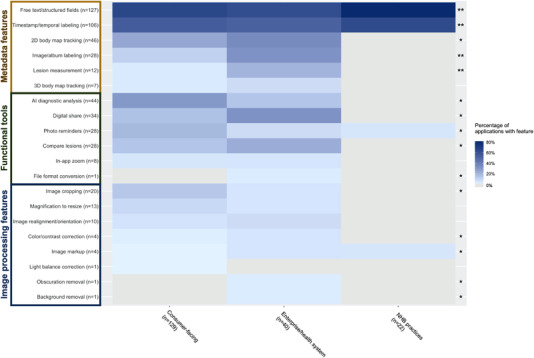
Feature frequency across skin imaging applications, by primary audience. (A) Heatmap of the frequency of individual features, relative to primary audience. Features are grouped and labeled by feature category on the *y*‐axis. Darker blue hues correspond to higher percentages and grey indicates 0%. In the rightmost column, * indicates *p*‐value <0.05 and ** indicates *p*‐value <0.01 for individual feature differences by audience category

The occurrence of individual features differed significantly by primary audience (*p* < 0.0001). Nearly all features were represented in skin imaging applications intended for consumer and enterprise/health system use, while only 4 of 20 features were present in NHB practice apps (Figure 2). Metadata features were highly represented across audience categories. Chi‐square residuals suggest that a strongly positive association between NHB practices and both text‐based and temporal labeling of skin images, as well as between enterprise/health system apps and both image/album labeling and lesion measurement functionality, was highly contributory to differences between audience categories. Functional tools and image processing features were represented within consumer and enterprise/health system apps, though functional tools were more common. AI‐based diagnostics and the ability to digitally share skin images occurred in over 30% of consumer and enterprise/health system applications, respectively. Notably, most features were positively associated with only one audience category.

**FIGURE 3:**
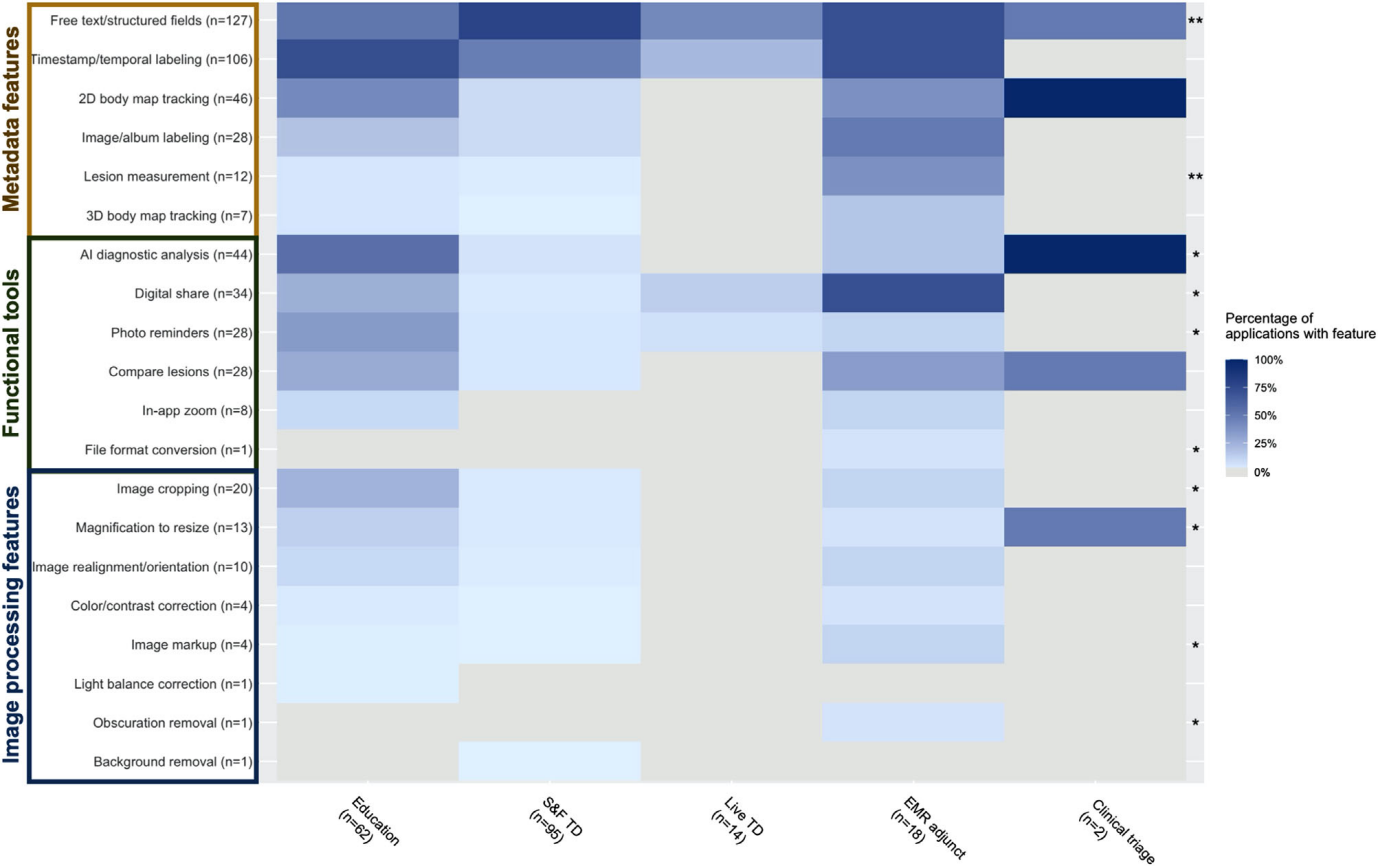
Feature frequency across skin imaging applications, by primary function. (A) Heatmap of the frequency of individual features, relative to primary function. Features are grouped and labeled by feature category on the *y*‐axis. Darker blue hues correspond to higher percentages and grey indicates 0%. In the rightmost column, * indicates *p*‐value <0.05 and ** indicates *p*‐value <0.01 for individual feature differences by primary function

Similarly, significant differences in feature occurrence were observed by primary function (*p* < 0.0001). Apart from text‐based and temporal labeling, which were common across all function groups, metadata features were most common in applications intended for educational use, S&F TD, and EMR/clinical imaging. Education and EMR/clinical imaging applications were also the most feature diverse, with the greatest representation of functional tools and image processing features (Figure 3). Over half of all education apps offered AI‐based diagnostics and more than 70% of EMR/clinical imaging tools enabled digital sharing. Interestingly, S&F TD applications were strongly positively associated with text‐based labeling while education apps showed a strong negative association. This trend reversed in many other features, though respective degrees of association were weaker. EMR adjunct tools were positively associated with lesion measurement and digital sharing features, while S&F TD was negatively associated with all functional tools.

On average, each application had just under three features. The mean number of total features per app, as well as the mean number of metadata and functional tools features only, varied significantly by primary audience and function (*p* < 0.001–0.05). While the number of image processing features did not vary significantly by audience, similar effects were seen for function and user type (*p* < 0.001 and *p* < 0.05, respectively). Interaction effects between audience and function groups were not significant in predicting total feature counts.

**FIGURE 4:**
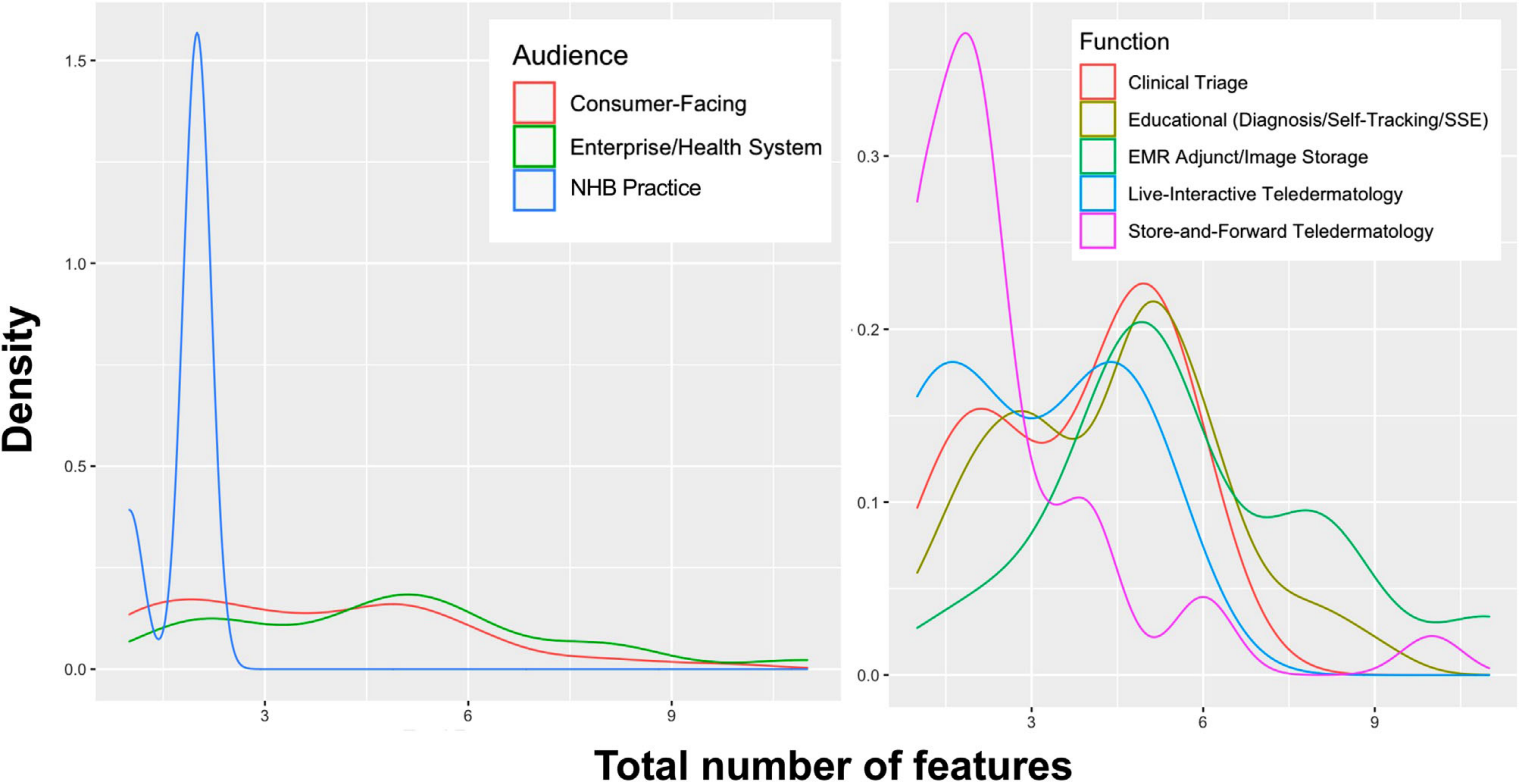
Density of total features per skin imaging application, by primary audience (left) and function (right). Total number of features are shown on the *x*‐axis and density on the *y*‐axis. Categories are denoted by line color, as described

Feature richness across feature types, defined here as the sum of individual feature counts, tended to be lowest in applications aimed at NHB practices and higher in those targeted to consumers and enterprise/health systems. Feature richness was also lower in applications used for S&F TD and close to uniformly higher in all other functions (Figure 4). Metadata feature counts were the exception, where both S&F TD and clinical triage applications had less than 1 metadata feature on average, while educational, EMR adjunct/image storage, and live‐interactive TD applications had roughly 2 metadata features on average. The most highly featured apps were *DermEngine* and *MoleScope*, with 11 and 10 features each.

At the time of data collection, only 25 (13%) skin imaging applications stated that they were HIPAA compliant in public marketing or informational materials. Less than half (45%) explicitly requested consent for the utilization of user‐uploaded photos, which we defined as requiring an active sign of agreement (i.e., checking a box or clicking “Yes” to proceed) on signup or before image submission. Fewer than 10% clearly described how images would be stored and handled after submission with respect to privacy concerns. Fifteen applications stated involvement in at least one clinical or regulatory study; both self‐reported and peer‐reviewed studies were included, regardless of data availability. Twelve applications described compatibility with EMR/EHR systems, though the majority did not specify which software were supported. Seven applications stated support of imaging standards such as DICOM and HL7.

## 4 DISCUSSION

Our findings suggest that image utilization features in digital skin imaging applications generally fall into three categories: metadata, functional tools, and image processing. Metadata features were nearly ubiquitous across primary audience and function categories, with many applications allowing users to provide additional information about uploaded skin images through free text/structured fields and temporal labeling. Functional tools’ features occurred in approximately half of applications and were intended for a variety of heterogenous functions. Despite the importance of image quality in many TD and self‐tracking applications, only one‐fifth of applications included any image processing features. Feature implementation was not standardized across applications, though several NHB practice apps appeared identical. Specific features including text‐based labeling, timestamps, lesion measurement, and digital sharing had large contributions to significant differences between audience‐ and function‐based categories.

Applications had an average of 2.7 total features each, with nearly two‐thirds having two or fewer features while only 12% had five or more. These data are consistent with previous studies that found low feature scores in acne management apps and substantial feature variability in dermatology patient education tools.^15^, ^16^ Per‐app feature counts across metadata, functional tools, and all feature categories demonstrated significant variation by primary application audience, function, and in limited cases, user type. Apps developed by NHB practices tended to be feature poor, whereas those aimed at consumers and enterprise/health system users had almost twice the mean number of features. Interestingly, apps that functioned as S&F and live‐interactive TD tools had fewer features than those intended for educational, EMR adjunct/clinical imaging, and clinical triage use. Given the centrality of provider assessment and management in TD, features that optimize the quality of submitted images may be more important than the post‐capture image utilization features described in this study.

Notably, 44 applications included some form of AI‐based diagnosis, defined as the use of computational technology to provide some form of clinical diagnosis as stated by the app creator. This was the most common functional tools feature, occurring more often than comparatively simple functionality such as the ability to set photo reminders or to share results between mobile apps. Diagnostic information was returned in multiple forms, some of which were occurred simultaneously within the same app. Most analyses returned the names of the most likely conditions, often in rank order or prioritized by a percent confidence score. Binary results were also common, where applications would indicate whether a lesion was malignant (skin cancer) or not. Several apps classed lesions as high, medium, or low risk and associated different risk classes with recommendations for future action (e.g., seeking out a general practitioner or specialist). In two cases, AI‐based diagnoses returned only a continuous risk score indicating the likelihood of melanoma. These scores were not clearly thresholded. Of the applications offering AI analysis, most did not describe how models were trained, disclose assessments of model performance, or specify which conditions could be diagnosed. Few gave clear instructions regarding the interpretation of results. The variability in our findings underscores the need for appropriate regulation and a more robust evidence‐base in AI‐based skin cancer detection.^33^, ^34^

Few applications addressed key health technology concerns such as regulatory (HIPAA) compliance, data storage, transmission, and encryption policies, or integration with existing EMR/EHR systems and medical imaging standards, which could preclude implementation in many clinical settings. Less than half explicitly asked users to consent to imaging, and those that did often neglected to specify how images would be used, how long they would be stored, or who would have access to them. Given the often‐identifiable nature of skin images and associated metadata, this lack of transparency poses non‐trivial privacy risks previously described in other mobile health apps.^35^, ^36^ A recent survey found that while less than 3% of patients using a secure mobile app for medical photography expressed privacy or confidentiality concerns, perceived acceptability of image re‐use declined as potential audience size increased.^37^ Our findings are also consistent with previous security assessments of mHealth apps more broadly, which highlight a lack of sufficient transport security measures and transparency in privacy regulations regarding user data.^38^, ^39^ Furthermore, little clinically relevant or published evidence supporting the use of individual applications could be identified. Only six apps have been described in published studies, though we found that over twice this number described self‐reported trial results.^14^, ^40^ To better evaluate claims of efficacy, data from unpublished studies should be made publicly available.

Limitations of our study include limited access to features, especially in the 70 (37%) apps that were paid, had paywalled features, or were enterprise apps, as well as variability in the purpose of skin imaging applications. Depending on intended usage, some features may be more important than others and feature richness (count) may not be an appropriate measure of relative app quality. Future directions can include prospective studies that evaluate the relationship between specific functionality and clinically relevant outcomes, as well as those aiming to develop standardized recommendations for patient consent and data storage in dermatology apps.

## 5 CONCLUSIONS

In summary, image utilization features in digital skin imaging applications generally relate to metadata, functional tools, or image processing. Apps display significant feature heterogeneity across primary audience and function categories, though those developed by NHB practices or intended for TD use tend to be less richly featured. Specific features such as text‐based labeling and digital sharing display association with specific categories. Many applications lack transparency regarding data storage and future use policies, as well as clinically relevant evidence that support their use. Improved regulation, especially of AI‐based apps, is needed for the widespread implementation of strategies to safeguard patient privacy and confidentiality.

## CONFLICT OF INTEREST

The authors received no specific funding for this work. The authors declare that there is no conflict of interest that could be perceived as prejudicing the impartiality of the research reported.

## Data Availability

The data that support the findings of this study are available from the corresponding author upon reasonable request.
